# Can the Non-pneumatic Anti-Shock Garment (NASG) reduce adverse maternal outcomes from postpartum hemorrhage? Evidence from Egypt and Nigeria

**DOI:** 10.1186/1742-4755-7-24

**Published:** 2010-09-01

**Authors:** Mohammed Mourad-Youssif, Oladosu A Ojengbede, Carinne D Meyer, Mohammad Fathalla, Imran O Morhason-Bello, Hadiza Galadanci, Carol Camlin, David Nsima, Tarek al Hussaini, Elizabeth Butrick, Suellen Miller

**Affiliations:** 1 Department of Obstetrics & Gynecology, El Galaa Maternity Teaching Hospital, Cairo, Egypt; 2 Center for Population and Reproductive Health, College of Medicine/University College Hospital, University of Ibadan, Ibadan, Nigeria; 3 Department of Obstetrics, Gynecology and Reproductive Sciences, University of California, San Francisco, California, USA; 4 Department of Obstetrics and Gynecology, Faculty of Medicine, Assiut University Women's Health Center, Assiut, Egypt; 5 Center for Population and Reproductive Health, College of Medicine/University College Hospital, University of Ibadan, Ibadan, Nigeria; 6 Aminu Kano Teaching Hospital, Kano, Nigeria; 7 Department of Obstetrics and Gynecology, Katsina General Hospital, Katsina, Nigeria

## Abstract

**Background:**

Postpartum hemorrhage (PPH) is the leading cause of maternal mortality and severe maternal morbidity. The Non-pneumatic Anti-Shock Garment (NASG), a first-aid lower-body compression device, may decrease adverse outcomes from obstetric hemorrhage. This article is the first to report the effect of the NASG for PPH.

**Methods:**

This pre-intervention/NASG study of 854 women was conducted in four referral facilities in Nigeria and two in Egypt between 2004-2008. Entry criteria were women with PPH due to uterine atony, retained placenta, ruptured uterus, vaginal or cervical lacerations or placenta accreta with estimated blood loss of ≥ 750 mL and one clinical sign of shock. Differences in demographics, conditions on study entry, treatment and outcomes were examined. The Wilcoxon rank-sum test and relative risks with 95% confidence intervals were calculated for primary outcomes - measured blood loss, emergency hysterectomy, mortality, morbidity (each individually), and a combined variable, "adverse outcomes", defined as severe morbidity and mortality. A multiple logistic regression model was fitted to test the independent association between the NASG and the combined severe morbidity and mortality outcome.

**Results:**

Measured blood loss decreased by 50% between phases; women experienced 400 mL of median blood loss after study entry in the pre-intervention and 200 mL in the NASG phase (p < 0.0001). As individual outcomes, mortality decreased from 9% pre-intervention to 3.1% in the NASG phase (RR 0.35, 95% CI 0.19-0.62); severe morbidity decreased from 4.2% to 1%, in the NASG phase (RR 0.24, 95% CI 0.09-0.67). As a combination, "adverse outcomes," decreased from 12.8% to 4.1% in the NASG phase (RR 0.32, 95% CI 0.19-0.53). In a multiple logistic regression model, the NASG was associated with the combined outcome of severe maternal morbidity and mortality (OR 0.42, 95% CI 0.18-0.99).

**Conclusion:**

In this non-randomized study, in which bias is inherent, the NASG showed promise for reducing blood loss, emergency hysterectomy, morbidity and mortality associated with PPH in referral facilities in Egypt and Nigeria.

## Background

Postpartum hemorrhage (PPH) is the single largest cause of maternal death worldwide. These deaths are largely preventable with skilled attendance and comprehensive emergency obstetric care. While the majority of maternal deaths from hemorrhage occur in low-resource settings, PPH-related mortality and morbidity are rising in higher-resource countries as well [1]. Developing improved strategies for managing PPH is essential to reducing these unnecessary deaths.

A woman suffering from PPH can die within 2 hours unless she receives immediate and appropriate medical care [2]. The identification of complications and the decision to take a woman to a health facility may be delayed and transportation may not be available. When a woman suffering from PPH arrives at a health facility, there may not be trained staff, essential supplies or medications available to treat her [3].

Uterine atony makes up the largest single etiology comprising PPH, and can be reduced by 50% with the performance of Active Management of Third Stage Labor (AMTSL) [4]. However, even with AMTSL, PPH from uterine atony can occur. Other etiologies of PPH, which are not prevented by AMTSL include retained placenta, ruptured uterus, vaginal/cervical lacerations and placenta accreta. These non-atonic PPH etiologies are not affected by the administration of uterotonics, and almost always require surgery. Current treatment protocols for PPH and hypovolemic shock include the administration of treatment uterotonics, bimanual massage, manual removal of the placenta, repair of lacerations, blood transfusion and surgery. All of these may be unobtainable in low-resource settings, except at tertiary facilities, and women may experience long delays receiving treatment even at these facilities [5].

**Figure 1:**
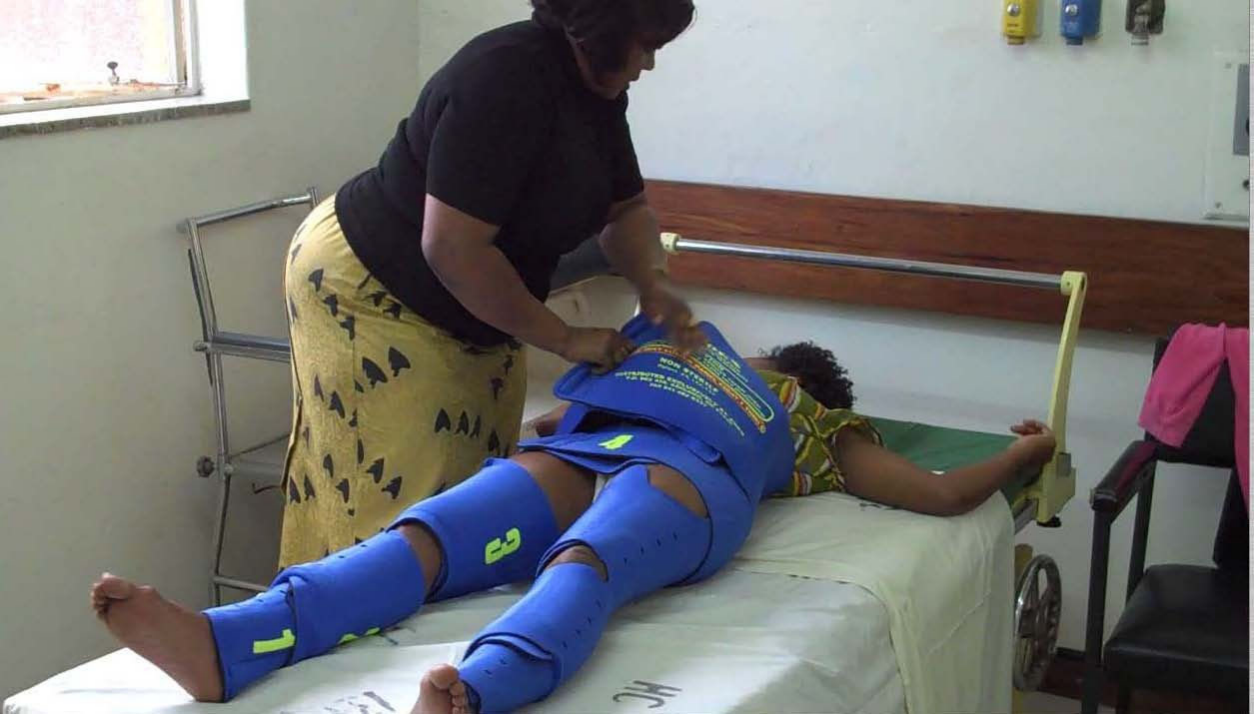
**An NASG fully applied**.

When delays in management of PPH occur, first-aid is needed to resuscitate and stabilize women with hypovolemic shock until definitive treatment is obtained. The Non-pneumatic Anti-Shock Garment (NASG) is a first-aid, lower-body compression device made from neoprene and Velcro™ (Zoex Corporation, Ashland, OR, USA). Each of its nine segments is sequentially wrapped tightly around a hemorrhaging woman's legs, pelvis and abdomen (Figure 1). The abdominal segment applies extra compression with a small foam ball. The circumferential counter pressure applied by the NASG reduces the total vascular space in the lower portion of the body while simultaneously increasing the volume of blood in the central circulation. Thus, oxygenated blood is shunted to the vital organs [6]. The application of the NASG reverses hemorrhagic shock and can stabilize a patient while awaiting transport, during transport, or during delays in receiving care at referral facilities.

The NASG is uniquely suited for use in low-resource countries due to its simple design and relatively low cost. Currently, the device costs $170 USD and training for application is relatively brief. After decontamination and laundering, the device can be re-used up to 40 times. Vaginal procedures are performed with the NASG in place and abdominal surgeries can be conducted by opening the abdominal section during surgery.

Published papers have reported the utility of the NASG for women with obstetric hemorrhage from all etiologies in low-resource referral facilities [7-11]. In this article, we examine the outcomes of the NASG on women experiencing PPH at two such facilities in Egypt and four in Nigeria. This sub-set of data on PPH etiologies has never before been explored, as PPH is the leading cause of obstetric hemorrhage, we feel it worthy of a separate analysis.

## Methods

This study is a sub-analysis of data collected between 2004 and 2008. The methods have been described in more detail in previously published studies of obstetric hemorrhage from all etiologies [11,12]. Briefly, this is a pre-intervention/intervention study, data were collected during the pre-intervention phase, when clinicians used a standardized obstetric hemorrhage protocol, for women with hypovolemic shock secondary to obstetric hemorrhage and outcomes of interest were recorded. Next, the NASG was introduced. During the NASG intervention phase, clinicians used the same protocol plus the NASG for women with hypovolemic shock secondary to obstetric hemorrhage. The pre-intervention and intervention phases were exactly the same length in the Egypt study, length of pre-intervention and intervention phases in Nigeria differed, but in each of the four facilities pre-intervention phases were shorter than intervention phases.

For the present PPH sub-set analysis, we examined only the data on women admitted for PPH (n = 854) at tertiary care facilities with roughly equivalent pre-intervention (n = 343) and NASG phases (n = 511). PPH diagnoses included uterine atony, retained placenta or tissue, ruptured uterus, vaginal/cervical lacerations and placenta accreta. To be eligible for the study, women presented with PPH and shock, defined by an initial blood loss of ≥ 750 mL and at least one clinical sign of shock (systolic blood pressure < 100 mmHg or pulse > 100 beats per minute).

The University of California, San Francisco (UCSF) Committee on Human Research (approval number H6899-23524), the National Reproductive Health Research Committee of the Nigeria Federal Ministry of Health, and the Ethics Committees of the El Galaa Maternity Teaching Hospital and Assiut University Women's Health Center gave ethical approval for the study. After an explanation of study aims, risks and benefits, all pre-intervention study participants gave verbal consent, and all NASG participants gave written consent to the use of the NASG. A United States Federal waiver of consent/authorization for minimal-risk research (45 CFR 46, 45 CFR 164.512) was obtained so that women who were unconscious or confused at the time of study entry were enrolled in the study without consent or with a relative's consent. Once the woman had regained consciousness and attained a normal sensorium, she was consented and gave written permission, even if a relative had given consent on her behalf while she was unconscious.

The evidence-based protocol for the treatment of PPH and shock included administration of crystalloid intravenous fluids (≥ 1500 mL in the first hour), uterotonic medications (oxytocin, ergometrine, syntometrine, misoprostol), uterine massage for patients with uterine atony, vaginal procedures (repair of lacerations, curettage for retained tissue, bimanual compression and/or manual removal of placenta) and when necessary, abdominal surgeries (arterial ligation, B lynch compression sutures, hysterectomy). After entry to the study, all study participants in both phases had blood loss measured with a calibrated blood collection and measurement drape (BRASS-V Fixable Drape™, Madurai, India), which was placed under them for accurate measurement of blood loss during resuscitation and hemorrhage treatment. This drape has been compared as equivalent to spectrophotometry for accuracy [13].

For women in the intervention phase of the study, the NASG was applied at the time of study admission and remained in place for the duration of the study. The NASG was applied sequentially, starting at the ankles with two segment #1 panels, one for each ankle, then to the calves (segments #2), thighs (segments #3), then segment #4 to the pelvis, and finally closing the double segment (#5 and #6), with the pressure ball, around the abdomen. All patients were monitored every 15 minutes for vital signs, level of consciousness, uterine condition, bleeding, and urine output, until they were stable. If surgery were required for NASG patients, the abdominal section was opened immediately before the skin incision and closed after the abdominal bandage was applied. Study discharge and NASG removal time were determined by two hours of hemodynamic stability (systolic blood pressure > 100 mmHg and pulse < 100 beats per minute) and vaginal bleeding of < 50 mL/hour. The device was then removed in the same order as it was applied, starting at the ankles with 15 minute intervals between each segment.

Clinician/data collectors were trained in the standard management of PPH and shock, collection and measurement of blood loss and completion of data collection forms. In Nigeria, paper data forms were reviewed by data supervisors and the Nigerian Principal Investigators, copied, and sent to UCSF where data were entered into a Microsoft Access database (Microsoft, Redmond, WA, USA) and checked for errors and inconsistencies. In Egypt, paper forms were reviewed by the Egyptian Principal Investigators and electronically transmitted by data fax (Clinical DataFax Systems Inc., Ontario, Canada) to UCSF.

A woman's condition on study entry was calculated using mean arterial pressure (MAP = [2*Diastolic Blood Pressure] + Systolic Blood Pressure/3) [14]. Primary outcomes included cumulative blood loss measured in the drape, emergency hysterectomy, and "adverse outcomes," which was a combined variable of severe maternal morbidity and mortality. Severe maternal morbidity included end-organ dysfunctions related to hemorrhage including cardiac, pulmonary, renal, and CNS [15]. Tests of significance of differences by study phase were chi-squares for categorical variables, t-tests (assuming unequal variances) for normally-distributed continuous variables and Wilcoxon rank-sum tests for non-normally distributed continuous variables. Normality was tested using *qnorm *and *sktest *in STATA.

To estimate the independent effect of the NASG, we used a multiple logistic regression model to control for other characteristics which might predict adverse outcomes. The independent variables included in the model were selected on the basis of their prior significance in the literature on maternal mortality and for significant association (at the 95% confidence level) with mortality and morbidity in bivariate analyses of this data. Variables included in the final model were high parity, MAP < 60, where the patient began bleeding (outside the facility and transferred in vs. began bleeding in the facility), and study phase (pre-intervention or NASG). All data were analyzed using STATA version 10 (StataCorp, College Station, TX, USA).

## Results

A total of 854 women were included in the analysis of which 343 were in the pre-intervention phase and 511 in the NASG phase. The demographic characteristics of the participants in the two phases are shown in Table 1. There were no statistically significant differences between the pre-intervention and NASG phases with respect to duration of pregnancy, parity, and most hemorrhage etiologies and conditions on study entry. Women in the pre-intervention phase were statistically younger (28.8 vs. 29.7, p = 0.042). There were more women with ruptured uterus in the pre-intervention, while there were more women with lacerations in the NASG phase. There were no differences in condition on study entry, except that more women in the pre-intervention phase were transferred in bleeding than in the NASG phase (51.4% vs. 29.1%, p < 0.001). Equal proportions, approximately 36%, had more severe shock, as measured by MAP < 60 mmHg.

**Table 1:** Demographics, diagnoses, and condition on entry to study (n = 854)

	Pre-intervention N = 343	NASG N = 511	*p *value
**Study Sites**			
Egypt (2 referral hospitals)	223	336	--
Nigeria (4 referral hospitals)	120	175	--

**Demographic Characteristics**			
Age: Mean years of age (SD)	28.8 (6.2)	29.7 (6.1)	0.042
Median age (IQR)	29 (25-33)	30 (25-35)	--
Parity: Mean parity (SD)	3.5 (3.0)	3.4 (2.6)	--
Median parity (IQR)	3 (1-5)	3 (2-4)	0.802
Pregnancy duration: Mean weeks (SD)	38.2 (2.8)	38.3 (2.6)	--
Median weeks (IQR)	39 (37-40)	39 (37-40)	0.814

**PPH Diagnoses**^€^			
Uterine atony	197 (57.4)	324 (63.4)	0.079
Vaginal, cervical or genital lacerations	24 (7.0)	65 (12.7)	0.007
Retained placenta or tissue	69 (20.1)	80 (15.7)	0.092
Ruptured uterus	45 (13.1)	32 (6.3)	0.001
Placenta accreta	8 (2.3)	10 (2.0)	0.809^√^

**Condition on Study Entry**			
Where hemorrhage began			< 0.001
Transferred in bleeding	145 (51.4)	104 (29.1)	
Began bleeding in hospital	137 (48.6)	253 (70.9)	
Estimated revealed blood loss at study entry^§^			
Mean mL (SD)	1223.8 (509.5)	1288.7 (447.9)	--
Median mL (IQR)	1000 (1000-1500)	1000 (1000-1500)	0.008
Women with MAP < 60 or non-palpable BP**	123 (35.9)	183 (35.9)	0.995

NASG = non-pneumatic anti-shock garment. Data are n (column %), mean (SD) or median (IQR). The denominator is the entire population, unless otherwise noted.

Tests of significance of differences by study phase were chi-square for categorical variables, t-tests (assuming unequal variances) for normally-distributed continuous variables and Wilcoxon rank-sum tests for continuous variables with non-normal distributions.

€PPH Diagnosis includes primary or secondary diagnosis of any of the following > 24 weeks with uterine atony, rupture, placenta accreta, vaginal/cervical lacerations, retained placenta or tissue.

√ Two-side Fisher's exact test used.

^§ ^Data missing for 37 patients.

** MAP < 60 category includes those with non-palpable blood pressure (BP). Data missing for 1 patient.

During both phases, women received comparable treatment as shown in Table 2, given the low-resource setting, many women in both phases experienced delays in receiving treatment. There were no statistically significant differences between administration of treatment uterotonics for women with a primary or secondary diagnosis of uterine atony, receipt of blood transfusion in the first hour or blood transfusion at any time during the study. There was a significantly higher proportion of women who received ≥ 1500 mL of IV fluids in the first hour in the pre-intervention group, but the proportion receiving fluids did not significantly differ by the second hour.

**Table 2:** Treatments for shock and hemorrhage during pre-intervention and NASG study phases (n = 854)

*Treatment*	Pre-intervention N = 343	NASG N = 511	*p *value
Any uterotonics administered*	215 (96.9)	343 (98.0)	0.414 ^√^
≥ 1500 mL IV fluids within 1st hour†	275 (80.2)	363 (71.0)	0.003
≥ 1500 mL IV fluids within 2nd hour†	300 (87.5)	453 (88.7)	0.599
Blood transfusion within 1^st ^hour	227 (66.2)	315 (61.5)	0.159
Blood transfusion after study admission	318 (92.7)	474 (92.8)	0.979

NASG = non-pneumatic anti-shock garment. Data are n (column %). The denominator is the entire population, unless otherwise noted.

*Of women with uterine atony as primary or secondary diagnosis. Data are for 572 cases.

^√ ^Two-sided Fisher's Exact test used.

† The protocol asked for 1500 mL to be administered in the first hour of resuscitation; the majority of women received fluids within the first hour and fifteen minutes.

Women in the NASG phase experienced better outcomes as demonstrated in Table 3. Mean measured blood loss decreased by 50% between phases. Women experienced a median of 400 mL blood loss after study entry in the pre-intervention and 200 mL in the NASG phase (p < 0.0001). There was a statistically significant decrease in the percentage of women who experienced an emergency hysterectomy for intractable uterine atony, 20 (9.0%) in the pre-intervention vs. 14 (4.0%) in the NASG (RR 0.44, 95% CI 0.23-0.86). For the combined outcome of severe morbidity and mortality, the results were also significantly better in the NASG phase, 44 (12.8%) in the pre-intervention vs. 21 (4.1%) in the NASG (RR 0.32, 95% CI 0.19-0.53). Individually, both severe morbidity and mortality were also significantly decreased in the NASG phase.

**Table 3:** Outcomes during pre-intervention and NASG study phases (n = 854)

*Outcome*	Pre-intervention N = 343	NASG N = 511	Relative Risk (95%CI)	*p*
Measured vaginal blood loss in drape*:				
Mean mL (SD)	424.1 (302.3)	220.5 (144.0)	--	
Median mL (IQR)	400 (250-500)	200 (200-210)	--	right< 0.0001
Emergency hysterectomy **^‡^**	20 (9.0)	14 (4.0)	0.44 (0.23-0.86)	--
Combined Outcome: Severe morbidity** and mortality	44 (12.8)	21 (4.1)	0.32 (0.19-0.53)	--
Morbidity**	13 (4.2)	5 (1.0)	0.24 (0.09-0.67)	--
Mortality	31 (9.0)	16 (3.1)	0.35 (0.19-0.62)	--

NASG = non-pneumatic anti-shock garment. Data are n (%) or mean (SD). The denominator is the entire population, unless otherwise noted.

* For cases in which the calibrated blood collection drape was used and there were data for blood loss. Wilcoxon rank-sum test used to compare distributions by study phase. Data are for 784 cases.

**^‡ ^**Data on emergency hysterectomy are only for women with primary or secondary diagnosis of uterine atony (n = 573).

** Includes renal failure, acute respiratory distress syndrome, heart failure, cerebral impairment (seizures, unconsciousness, motor/cognitive loss) lasting more than 24 hours after resuscitation from shock. Denominator is the number of women who survived (n = 807).

In the bivariate analyses (simple logistic regression) the following variables, in addition to study phase, were found to be significantly associated with mortality at the 95% confidence level: parity, severity of shock, and where bleeding began (outside of facility and transferred in vs. began bleeding in facility). Using a multiple logistic regression model that included these variables, we estimated the independent association between the NASG intervention and the combined outcome variable (mortality and severe morbidity). In the multiple regression model, as shown in Table 4, severity of condition upon study admission was strongly associated with mortality after controlling for other variables in the model. Those women with MAP < 60 mmHg had 19 times the odds of suffering the combined adverse outcomes variable (adjusted odds ratio (aOR) 19.1, 95% CI 6.95-52.65, p < 0.001). High parity and where bleeding began were not significantly associated with mortality/morbidity in the adjusted model. The NASG remained significantly associated with reduced odds of adverse outcome, (aOR 0.42, 95% CI, 0.18-0.99, p = 0.046).

**Table 4:** Multiple logistic regression models of factors predictive of combined outcome severe maternal morbidity and mortality (n = 639)

Factor	Dependent variable: Combined Severe Morbidity and Mortality			
	
	Adjusted OR	*p*	95% CI	
**Severity of Shock**				
MAP < 60 (or non-palpable BP)	19.1	< 0.001	6.95	52.65
*MAP 60 or higher*	*1*			
**Parity**				
5 or more live births	2.29	0.050	1.00	5.26
*0-4 live births*	*1*			
Where Bleeding Began*				
Transferred in bleeding	1.88	0.222	0.68	5.15
*Began bleeding in hospital*	*1*			
**Study Phase**				
NASG	0.42	0.046	0.18	0.99
*Pre-intervention*	*1*			

NASG = non-pneumatic anti-shock garment, MAP = Mean Arterial Pressure, BP = blood pressure. Reference groups for categorical variables shown in italics. The number of observations in Table 4 is less than 854 because of missing data. Hospital facility was used as a control variable in the model but not shown in Table 4.

Because of the very strong association of severity of condition (MAP < 60) with mortality and morbidity, we ran a stratified analysis of the effect of MAP < 60 on outcome by phase. In this analysis the NASG significantly improved outcomes for those with both more and also less severe shock. The odds of extreme adverse outcome were 0.19 (95% CI 0.02 - 0.99) for the MAP > 60 group and 0.27 (95% CI 0.14-0.52) for the MAP < 60 group. The NASG was significantly associated with reductions in adverse outcome for both groups, and there were no statistical differences between the odds in the two groups (data not shown).

## Discussion

PPH, a life-threatening condition globally, is associated with 25% of maternal deaths [1]. Adding the NASG to a standardized PPH and hypovolemic shock management protocol in tertiary facilities in Egypt and Nigeria was associated with a significant reduction in blood loss, emergency hysterectomy, combined mortality and severe morbidity, and for morbidity and mortality individually, even when controlling for severity of shock at study entry, parity, and where the woman began bleeding.

The NASG is not therapy or treatment but it can be used to buy time to obtain definitive treatment. Women in the NASG phase of the study still experienced blood loss, emergency hysterectomies, mortality, and severe morbidities, but at a lower rate than women who did not receive the NASG, regardless of condition on study entry or timing of treatment given. Rapid administration of blood, crystalloid fluids, uterotonics, and access to anesthesia and surgery are responsible for saving the lives of women with PPH; the NASG enables women to better survive delays until they receive these crucial treatments.

This study is limited primarily by the non-randomized, pre/post-intervention design, which includes the possibility of selection bias, which may have occurred as eligibility criteria and clinicians influence limited entry into the study. However, there were few statistically significant differences in demographics and condition on study entry between the two phases and the regression analysis was conducted to reduce the effect of differences between the phases. Another limitation is associated with the lack of temporal phase concurrence. The study clinicians' skill levels may have improved over time, as a result of the frequent trainings and more diligent practice of the evidence-based protocol. However, fewer women in the NASG received the protocol of > 1500 mL crystalloid IV fluid during the first hour. Further, research in under-staffed and over-burdened hospitals in low-resource settings could result in lack of protocol adherence, for example, fewer than 70% of all women received a blood transfusion in the crucial first hour of shock resuscitation. It is also possible that some women who were referred for severe hemorrhage died before reaching the facility, so we may be underestimating the mortality rates. The length of the pre-intervention phase was shorter than the intervention phase in each of the four Nigerian facilities, this partially accounts for the larger number of women in the intervention phase; however, there were no demographic differences or differences in condition on study entry between women in the two phases.

Statistical analyses were limited by the rare outcomes of morbidity and mortality. Odds ratios may be inflated in small studies, and it is possible that they are inflated in this study given that adverse outcome events were rare. Corrections to adjust for rare event bias (e.g. alternatives to maximum likelihood estimators) have been suggested in the literature, but the slight gains in precision afforded were not preferred due to the increased complexity of the results. Thus, we opted for a conventional statistical test (multiple logistic regression) that can be easily interpreted by a wide readership.

## Conclusion

The NASG may be useful in reversing shock and buying time, thereby reducing adverse outcomes from PPH at the referral facility level. While advances in AMTSL, prophylaxis with uterotonics, and enhanced treatment of uterine atony with uterotonics, including misoprostol, are now being stressed [4,16,17], not all uterine atony will respond to these measures. Further, uterotonics will not address non-atonic etiologies such as lacerations or ruptured uterus. In this study, uterine atony comprised 57.4% (pre-intervention phase) and 63.4% (NASG phase) of all PPH etiologies, thereby excluding 40% of women with PPH from rapid medical treatment. Balloon condom tamponade is suggested for PPH, but again, it is a treatment for uterine atony, and, as with the uterotonics, does not address hypovolemia [18,19]. In many low-resource settings there is currently no treatment for hypovolemic shock during long delays prior to obtaining definitive treatment.

The NASG's simple and inexpensive design makes it an easy to use first-aid device in low-resource referral facility settings. These findings show promise for saving women's lives from PPH and add to a growing NASG literature for all etiologies of obstetric hemorrhage in Pakistan, Mexico, Egypt and Nigeria [9-12].

New strategies and technologies are needed to reduce the global public health epidemic of maternal mortality. This study indicates the potential of the NASG at the referral facility for PPH. However, many women birth at home attended by unskilled birth attendants or family members. Therefore, attention should be focused on evaluation of the use of the NASG at the clinic level to determine if earlier application, before and during transport, will have a greater impact on decreasing adverse maternal outcomes.

## Competing interests

The authors declare that they have no competing interests.

## Authors' contributions

MMY participated in the conception of the study, acquired data, administered and managed the study, and helped draft the manuscript. OAO acquired data and administered and managed the study. CM helped draft the manuscript and performed statistical analyses. MF participated in the conception of the study, acquired data, administered and managed the study, and helped draft the manuscript. IOM acquired data and administered and managed the study. HG acquired data and administered and managed the study. CC performed statistical analyses. DN acquired data and administered and managed the study. EB administered and managed the study. SM conceived of the study, obtained funding, participated in study design and coordination, acquired data and drafted the manuscript. All authors read and approved the final manuscript.
